# Neuropsychiatric manifestations of COVID-19 can be clustered in three distinct symptom categories

**DOI:** 10.1038/s41598-020-78050-6

**Published:** 2020-12-01

**Authors:** Fatemeh Sadat Mirfazeli, Atiye Sarabi-Jamab, Amin Jahanbakhshi, Alireza Kordi, Parisa Javadnia, Seyed Vahid Shariat, Oldooz Aloosh, Mostafa Almasi-Dooghaee, Seyed Hamid Reza Faiz

**Affiliations:** 1 Mental Health Research Center, School of Behavioral Sciences and Mental Health (Tehran Institute of Psychiatry), Iran University of Medical Sciences, Tehran, Iran; 2 School of Cognitive Sciences, Institute for Research in Fundamental Sciences (IPM), P. O. Box 19395-5746, Tehran, Iran; 3 Department of Neurosurgery, Skull Base Research Center, Iran University of Medical Sciences, Tehran, Iran; 4 Faculty of Medicine, Iran University of Medical Sciences, Tehran, Iran; 5 Department of Internal Medicine, School of Medicine, Rasoul Akram Hospital, Iran University of Medical Sciences, Tehran, Iran; 6 Neurology Department, Firoozgar Hospital, Iran University of Medical Sciences, Tehran, Iran; 7 Rasoul Akram Hospital Clinical Research Development Center (RCRDC), Iran University of Medical Sciences, Sattarkhan St, Tehran, Iran

**Keywords:** Psychology, Diseases, Health care, Medical research

## Abstract

Several studies have reported clinical manifestations of the new coronavirus disease. However, few studies have systematically evaluated the neuropsychiatric complications of COVID-19. We reviewed the medical records of 201 patients with confirmed COVID-19 (52 outpatients and 149 inpatients) that were treated in a large referral center in Tehran, Iran from March 2019 to May 2020. We used clustering approach to categorize clinical symptoms. One hundred and fifty-one patients showed at least one neuropsychiatric symptom. Limb force reductions, headache followed by anosmia, hypogeusia were among the most common neuropsychiatric symptoms in COVID-19 patients. Hierarchical clustering analysis showed that neuropsychiatric symptoms group together in three distinct groups: anosmia and hypogeusia; dizziness, headache, and limb force reduction; photophobia, mental state change, hallucination, vision and speech problem, seizure, stroke, and balance disturbance. Three non-neuropsychiatric cluster of symptoms included diarrhea and nausea; cough and dyspnea; and fever and weakness. Neuropsychiatric presentations are very prevalent and heterogeneous in patients with coronavirus 2 infection and these heterogeneous presentations may be originating from different underlying mechanisms. Anosmia and hypogeusia seem to be distinct from more general constitutional-like and more specific neuropsychiatric symptoms. Skeletal muscular manifestations might be a constitutional or a neuropsychiatric symptom.

## Introduction

In December 2019 a number of severe acute respiratory syndrome (SARS) were reported in Wuhan, China that became eventually a pandemic infection with over 8 million reported cases until June 2020^1^. The typical symptoms include fatigue, fever, cough, and diarrhea. The infective agent, a coronavirus SARS-CoV-2, was named COVID-19 by World Health Organization (WHO) on 11 February 2020 which is a human coronavirus. Six other corona viruses are introduced that are hosted by humans: MERS-CoV, SARS-CoV, HCoV-229E, HCoV-HKU1, HCoV-NL63, and HCoV-OC43. COVID-19 is believed to attack respiratory and gastrointestinal systems (GI) by attaching to angiotensin-converting enzyme 2(ACE2) receptors^2,3^.

It has been documented that the β-coronaviruses like COVID-19 tend to involve the central nervous system, mainly brainstem. The ACE2 receptors in the brain are believed to play a role^3,4^. A direct route of entry into the central nervous system through the olfactory nerve is also postulated^5^. This finding is consistent with the prevalent and early olfactory symptoms in COVID-19^6^. However, it is not yet clear whether all patients with olfactory symptoms would develop neurologic complications, or all patients with neurological complications would show olfactory symptoms. Most studies on neurologic manifestations utilized frequency tests^6,7^ while using more advanced analysis such as clustering analysis would help us to furtherly categorize the neuropsychiatric manifestations.

Neurological manifestations has been reported up to 36% of patients in one study on 214 COVID-positive patients in Wuhan^7^. Encephalitis and meningitis are also rarely reported as a complication of COVID-19^8,9^. However, our knowledge is still scarce regarding characteristics of neurologic symptoms; whether or not they could be a sign of more severe case of COVID-19 or there is any correlation with other prognostic biomarkers such as lactic dehydrogenase (LDH), lymphocyte and high-sensitivity C-reactive protein (hs-CRP)^10^, or other clinical and paraclinical presentations.

Few studies have evaluated the neuropsychiatric manifestations of COVID-19 and to our knowledge; none of them have used a statistical clustering method to assess the symptom clusters of these manifestations. Identifying the clusters of related clinical findings is of great importance, because different clusters might be caused by distinct underlying mechanisms. Therefore, we decided to investigate the neuropsychiatric findings in COVID-19 patients by using a hierarchical clustering method in one of the largest referral centers in Iran.

## Results

**Figure 1:**
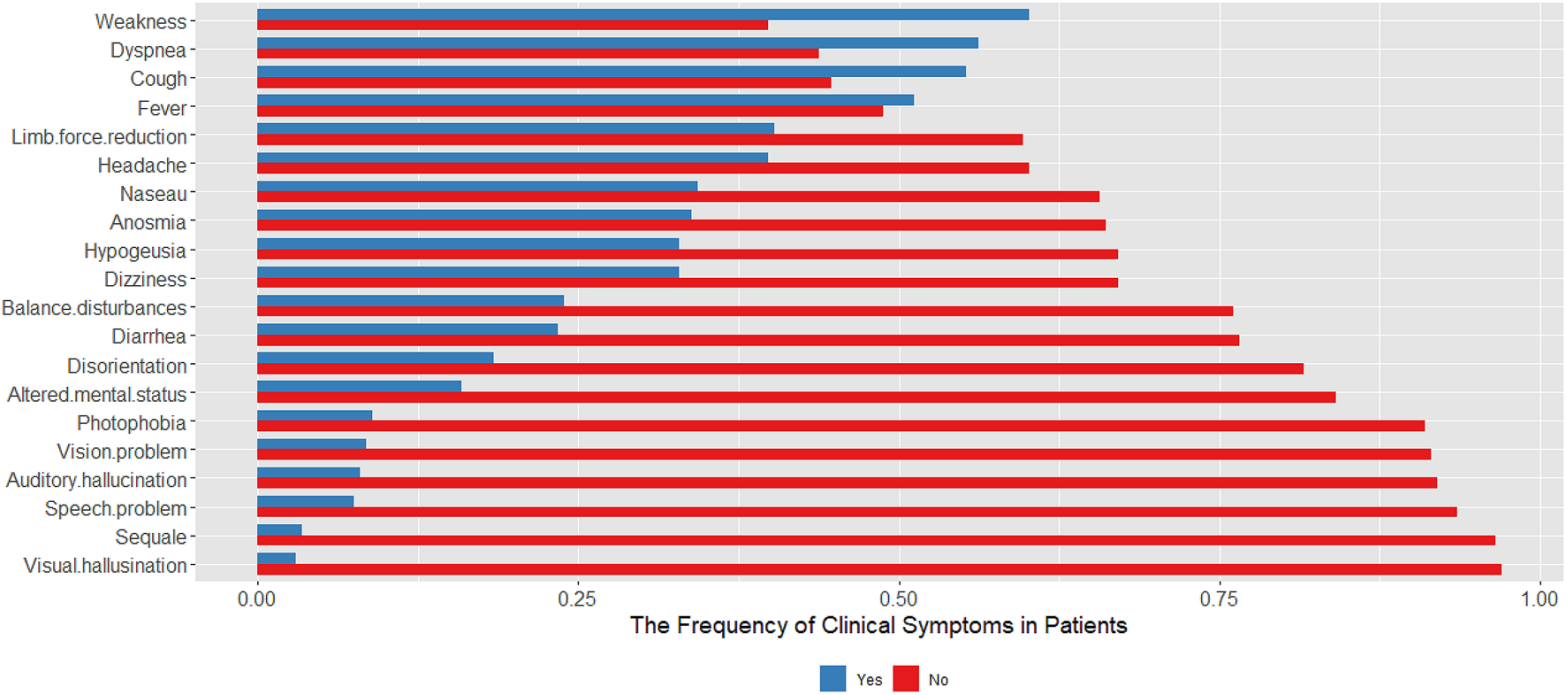
The frequency of neuropsychiatric manifestations along with respiratory and gastrointestinal symptoms in 201 patients with COVID-19.

Muscle weakness (60.2%) was the most common symptoms followed by dyspnea (56%), cough (55.2%), and fever (51.2%).One hundred and fifty-one patients (75.1%) showed at least one neuropsychiatric symptom. Limb force reduction (40.3%), followed by headache (39.8%), anosmia (33.8%), hypogeusia (32.8%) were among the most common neuropsychiatric symptoms in COVID-19 patients (Fig. 1).

### The relation between the neuropsychiatric manifestations with other clinical symptoms

To investigate the clinical symptoms in COVID-19 patients, we used a hierarchical clustering approach for identifying different groups of symptoms. This approach resulted in an attractive tree-based representation of the observations, called a dendrogram. In this approach each object is initially considered as a single-element cluster, and at each step of the algorithm, the two clusters that are the most similar are combined into a new bigger cluster. This procedure is iterated until the tree of the cluster is generated. To measure the dissimilarity, we used Gower dissimilarity that can be used to calculate the distance between two entities whose attribute has categorical values. The dissimilarity between two clusters is calculated based on minimizing the total within-cluster variance.

The result of our hierarchical analysis showed that we have six main clusters. Neuropsychiatric symptoms grouped together in three distinct groups: anosmia and hypogeusia (olfactory symptoms); dizziness, headache, and limb force reduction (general constitutional-like neuropsychiatric symptoms); photophobia, mental state change, hallucination, vision and speech problem, seizure, stroke and balance disturbance (specific neuropsychiatric symptoms or specific CNS type). Three non-neuropsychiatric cluster of symptoms included diarrhea and nausea (gastrointestinal symptoms); cough and dyspnea (respiratory symptoms); and fever and weakness (constitutional symptoms). It seems that the cluster of GI symptoms is completely separated from cluster of respiratory symptoms while it is closer to the neurological symptoms (specifically the ones with olfactory and general constitutional-like symptoms). However, cluster of constitutional symptoms is closer to the cluster of respiratory symptoms. Skeletal muscular manifestations might be a constitutional or a neuropsychiatric symptom.

**Figure 2:**
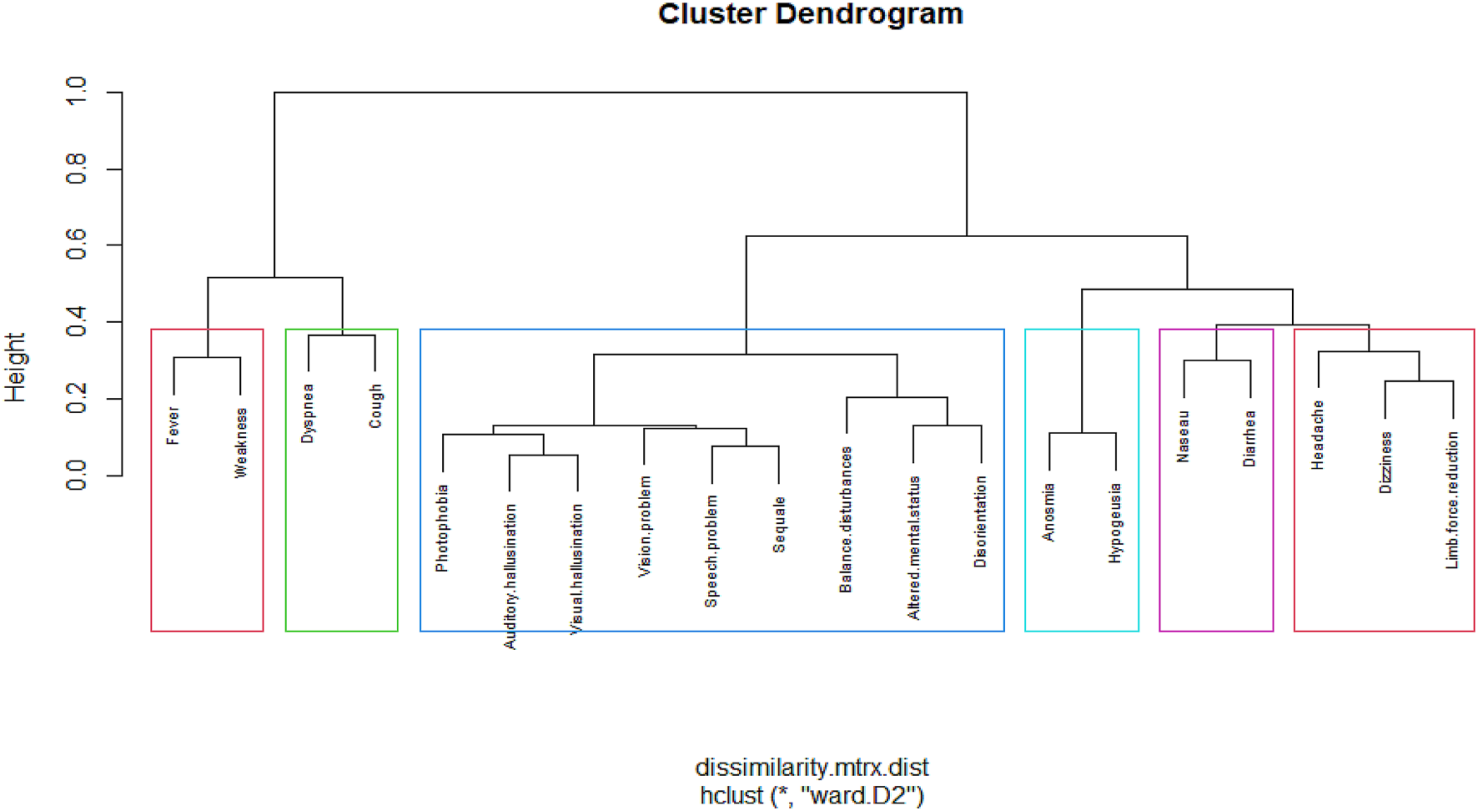
Tree-based representation (clustering) of the clinical symptoms in 201 patients with COVID-19 showing six main clusters: (olfactory symptoms), (general constitutional-like neuropsychiatric symptoms), (specific neuropsychiatric symptoms or specific CNS type), (gastrointestinal symptoms), (respiratory symptoms), (constitutional symptoms). In this figure the Sequel means seizure and stroke.

Figure 2 shows the cluster dendrogram of the clinical symptoms.

**Figure 3:**
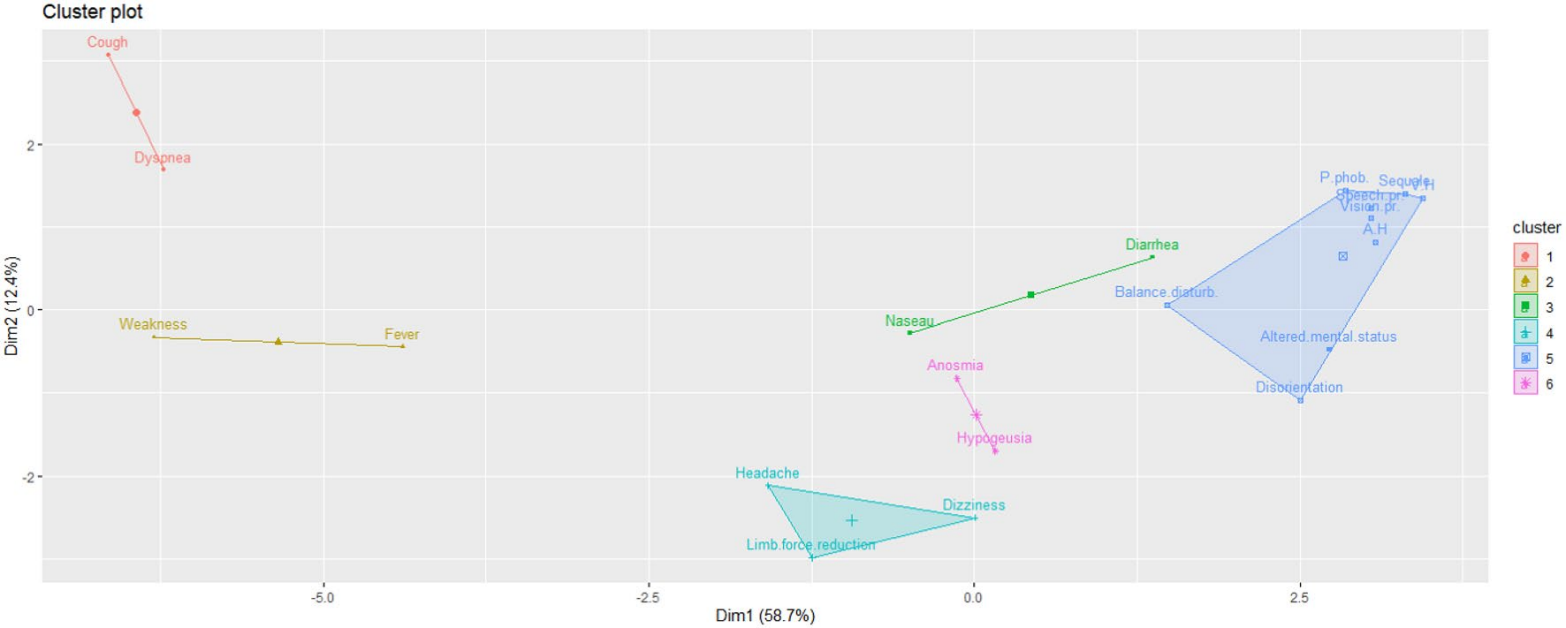
The scatter plot of the relation between neuropsychiatric symptoms and respiratory symptoms, gastrointestinal symptoms and constitutional symptoms in 201 patients with COVID-19.

Moreover, to visualize clearer, the relation between neuropsychiatric symptoms and other clinical symptoms is shown in Fig. 3 as a scatter plot.

### Laboratory measure findings in patients with COVID-19

**Figure 4:**
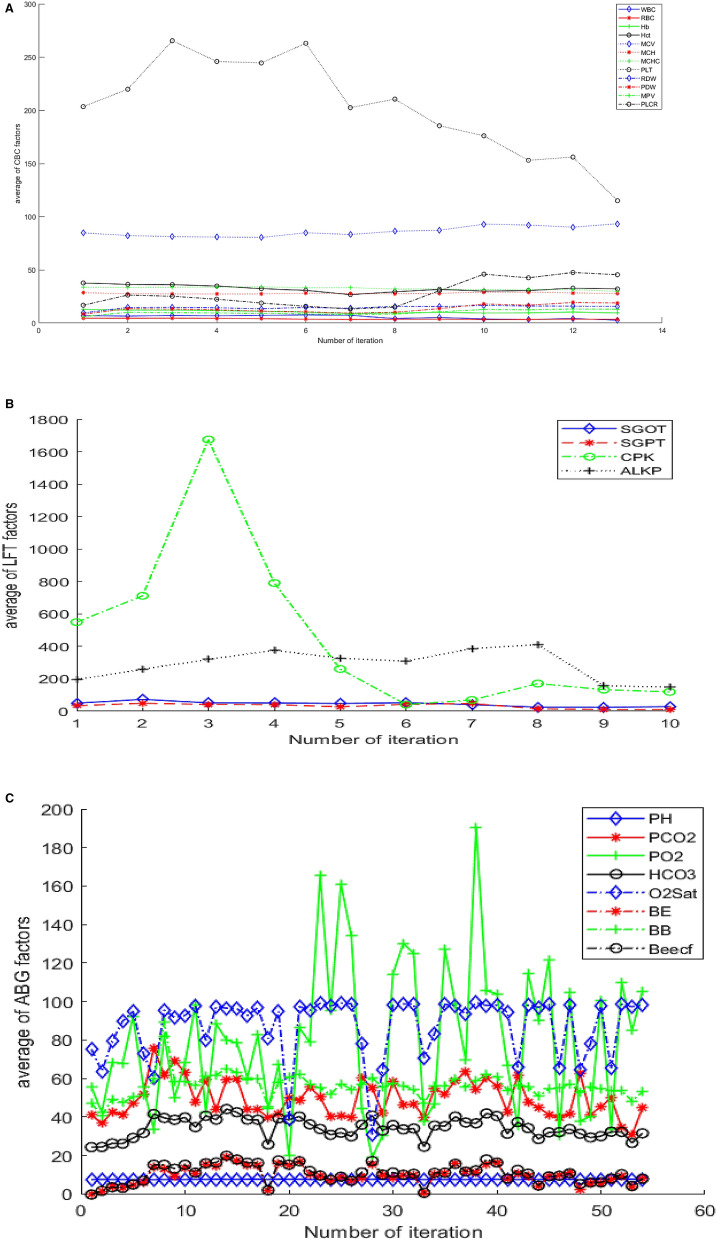
The trend of CBC, LFT, and ABG tests on 20 (**A**,**B**) and 10 (**C**) hospitalized patients with COVID-19. (**A**) The trend of CBC factors in 20 patients with COVID-19. The result shows a decrease following the increase of PLT count as the disease progresses. (**B**) The trend of LFT and CPK in 20 patients with COVID-19. There is a surge of CPK in the third testing, (**C**) The trend of ABG factors in 10 hospitalized patients with COVID-19.

CBC, LFT, and ABG tests were requested for some hospitalized patients and these tests were repeated if necessary, in some cases. To know about the trend of changing on these tests, we took the average measures based on the number of patients that are tested on that iteration. Figure 4A, B, C shows the trend of change in CBC, LFT, and ABG test, respectively.

We also investigated which factors of demographic factors such as age, gender, and clinical presentation could be the best predictors of WBC, RBC, and Platelet results. Therefore, we used a stepwise selection method to find the best subset of predictors.

The results of linear regression for finding the predictors of WBC count are shown in Table1. These variables jointly explain 79% variance of scores, R^2^ = 0.79, F (19, 13) = 2.69, *P* value: 0.03.

**Table 1:** Linear regression analysis showing predictors of WBC count, SGPT, SGOT in 20 patients with COVID-19.

Coefficients	Estimate std	Error	t value	Pr.( >|t|)
**WBC predictors**				
Intercept	9.81	2.07	4.73	< 0.001***
Gender male	− 2.29	1.58	− 1.44	0.17
Onset	− 0.20	0.07	− 2.61	0.02*
Dyspnea	3.96	1.56	2.53	0.02*
Cough	− 3.66	1.40	− 2.61	0.02*
Nausea	− 4.12	1.54	− 2.66	0.01*
Diarrhea	5.77	1.79	3.21	0.006**
Headache	1.38	1.45	0.94	0.36
Balance disturbance	− 8.83	2.17	− 4.05	0.001**
Speech problem	− 5.90	3.57	− 1.65	0.12
Weakness	2.72	1.42	1.90	0.07
Limb force reduction	− 4.01	1.93	− 2.07	0.05
Auditory hallucination	− 13.27	3.65	− 3.63	0.003**
Visual hallucination	7.98	4.31	1.85	0.08
Altered mental status	2.54	2.31	1.10	0.29
Sequel (stroke and seizure)	− 2.64	2.69	− 0.98	0.34
Anosmia	4.43	1.77	2.50	0.02*
Hypogeusia	− 7.28	2.51	− 2.89	0.01*
Vision problem	3.63	2.76	1.31	0.21
Disorientation	14.75	3.20	4.60	< 0.001***
**SGPT predictors**				
Intercept	61.98	15.46	4.008	< 0.001***
Cough	− 33.46	17.36	− 1.92	0.06
Fever	46.18	18.24	2.53	0.01*
Balance disturbances	− 46.68	26.77	− 1.74	0.09
Limb force reduction	− 53.49	20.35	− 2.62	0.01*
Auditory hallucination	178.23	54.28	3.28	0.002**
Visual hallucination	− 154.01	60.96	− 2.52	0.01*
Altered mental status	− 44.33	28.77	− 1.54	0.13
Anosmia	− 105.16	31.11	− 3.38	0.002**
Hypogeusia	110.00	35.90	3.06	0.004**
Disorientation	61.74	39.67	1.55	0.13
**SGOT predictors**				
Intercept	68.11	17.20	3.95	< 0.001***
Photophobia	171.89	61.23	2.80	0.008**

*p value < 0.05,**p value < 0.01,***p value < 0.001.

The results of linear regression for finding the predictors of SGPT are also shown in Table 1. These variables explain 59% variance of scores, R^2^ = 0.59, F (10, 27) = 2.69, *P*-value: 0.02. Furthermore, the results of linear regressing for finding the predictors of SGOT shows that only photophobia explains 17% variance of scores, *R*^2^ = 0.17, F (1, 36) = 2.69, *P* value: 008, see Table 1.

## Discussion

To the best of our knowledge, this study is one of the fewest attempts to explore and categorize neuropsychiatric manifestations along with other organ involvements in COVID-19. Hierarchical clustering analysis showed that neuropsychiatric symptoms group together in three distinct groups: anosmia and hypogeusia (olfactory symptoms); dizziness, headache, and limb force reduction (more constitutional-like neuropsychiatric symptoms); photophobia, mental state change, hallucination, vision and speech problem, stroke, seizure and balance disturbance (more specific neuropsychiatric symptoms or more specific CNS-type). Three non-neuropsychiatric cluster of symptoms included diarrhea and nausea (GI symptoms); cough and dyspnea (respiratory symptoms); and fever and weakness (constitutional symptoms). It seems that the cluster of GI symptoms is completely separated from the cluster of respiratory symptoms while it is closer to neurological symptoms (specifically the ones with olfactory and general constitutional-like symptoms). However, cluster of constitutional symptoms is closer to the cluster of respiratory symptoms. Having all serious neurological symptoms in one cluster is a probable sign for a different type of COVID-19 (maybe specific CNS type).

Neuropsychiatric manifestations were very prevalent among our patients (75.1%). They showed a variety of neuropsychiatric complications from limb force reduction (40.3%) and headache (39.8%) as the most common symptom and seizure (2%) and stroke (1%) as a rare consequence of COVID-19. These findings are almost in line with previous studies^7^, however in Ling Mao et al. study, fewer patients (36.4%) were reported to show neuropsychiatric complications^7^. This discrepancy could be explained by several reasons, one of them might be the fact that we had a more comprehensive checklist and we included symptoms such as hallucination, speech problems, photophobia in our checklist. The other reason would be Ling Mao et al.^7^ reported muscle injury while we documented decreased muscle force irrespective of the range of CPK. Consistent with the study of Ling Mao et al.^7^ seizure and stroke were one of the rarest presentations in CNS involvement of covid-19. Stroke as a sequel of COVID-19 came up in other studies^11^, as well. In one study in Italy, they reported a 2.5% ischemic stroke in COVID-19 patients^11^. In comparison in China among hospitalized COVID-19 patients, this rate was around 5%^12^. These slight differences might be due to different sampling, different screening tools, and other methodological distinctions. However, it is not yet explored which patients would present with such serious squeals.

Around 11% of our patients reported visual or auditory hallucination which could be secondary to delirium, or neuro-inflammation, both of which are possible based on previous literature^13,14^. However, the second one seems more probable in our patients because although hallucination and disorientation are in the same cluster, hallucination was closer to other neuropsychiatric presentations rather than disorientation. In Ferrando et al. study, psychosis was reported in three alert and oriented patients as a direct result of COVID-19 CNS involvement^13^.

In 9% of our patient photophobia was detected. Even though photophobia is not a common presentation, having it in a cluster with altered mental status and other neurological symptoms can imply the possibility of encephalitis or aseptic meningitis. Case reports of encephalitis have been already reported in these patients^15^. However, other etiologies such as medication side effects for photophobia should be kept in mind.

Altered mental status from disorientation to complete loss of consciousness has been seen in the patients which can be caused by several factors such as electrolyte disturbances, hypoxia, delirium, encephalopathy and stroke^15^. Due to possibility of CNS involvement and multi-organ damage we should consider a list of differential diagnosis in approaching the COVID-19 patients with mental status change rather than attributing it to just hypoxic encephalopathy. However, our knowledge is still scarce regarding the underlying mechanism of mental state change in COVID-19 patients. Different efforts have been made to explain underlying mechanisms of mental status changes and other neuropsychiatric presentations of COVID-19. Receptor injury, cytokine storm, retrograde travel via nerve fibers, secondary damage due to hypoxia have all been suggested as possible mechanisms^9^. The COVID-19 virus is believed to attach ACE receptor in GI, in epithelial cells of the lungs, in endothelial cells of blood–brain-barriers leading to cytokine release and inflammatory responses in the targeted organs^2,3,16–18^. This will cause neuro-inflammation along with lung and other organ damages. Alveolar damage may further lead to hypoxia and possible ischemic neural damage as a result. It has been also proposed that the virus might travel ascendantly via the olfactory bulb to the brain, creating a connection between nasal epithelium and central nervous system^19^. Accordingly, about one third of the patients presented anosmia and hypogeusia but these two symptoms shared a cluster together, rather than with the rest of neurologic symptoms. It may be another implication that there is more than one mechanism for CNS involvement (i.e., via retrograde transmission through olfactory bulb). Yet it is not clear in which patient every one of these mechanisms may activate. However, consistent with other studies^20^, the rate of anosmia and hypogeusia, were higher than Ling Mao et al. report^7^. This can be another reason for higher rate of neuropsychiatric manifestations in our findings.

Moreover, skeletal muscular manifestations in COVID-19 clustered in two distinct groups, one was subjective muscle weakness which clustered with constitutional symptoms of fever and the other was decreased force of limbs which clustered with two other neuropsychiatric symptoms (dizziness and headache). It may show two distinct underlying mechanisms for neuromuscular manifestations. We also had a surge of CPK in the trend of laboratory measure in our findings. However due to small sample size it was not clear which symptoms would represent actual muscular injury.

Added to finding the proximity of clinical symptoms, we also found an association between some clinical presentations and biomarkers such as WBC, SGPT, and SGOT. In recent articles role of biomarkers such as WBC, PLT, lymph count, and IL6 factor in disease risk stratification have been investigated^21^.

Finding heterogeneous neuropsychiatric manifestations from mild to severe conditions in a notable proportion of patients necessitates the neuropsychiatric examinations in all COVID-19 patients. Clustering these neuropsychiatric presentations in three distinct symptom categories besides providing some possible underlying pathophysiological mechanism for the disease, have also the following clinical implications as well:

1. It suggests that in the thorough neuropsychiatric assessment of the patients with COVID-19, all the three neuropsychiatric symptom categories should be covered.
2. Importance of each of these categories seems to be different. For example, finding a specific neuropsychiatric symptom might be a red flag mandating immediate further investigation such as neuroimaging. However, anosmia does not seem to be associated with more grave condition.
3. Patient who has symptoms in different categories might need various treatment options. The more general constitutional-like symptom category might only need hydration and resting .The specific neuropsychiatric symptom category, however might need aggressive treatment such as plasmapheresis^22^ or corticosteroid therapy^23^.

This study was not without limitations. We should consider that some of these neuropsychiatric presentations might be due to reasons other than coronavirus 2 such as medication side effect, electrolyte disturbance, hypoxia, comorbid diseases. It was a cross-sectional study and only laboratory tests with a clinical indication were requested for the patients. Larger sample sizes with larger laboratory measures could show a better picture of the disease. Having the genotype of the virus and HLA mapping of the patients in combinations with their clinical clustering and para-clinical presentation could help us understand the various behavior of the virus better and come closer to find a treatment. Brain imaging in our study was only ordered for critical patients and may some patients with stroke gone under diagnosed amid the crisis. Considering COVID-19 virus as a pathogen with the possibility of nervous system invasion will help clinicians to suspect COVID-19 in patients with CNS symptoms and to avoid misdiagnosis of COVID-19.

Neuropsychiatric presentations are very common and heterogeneous in patients with COVID-19 and should be evaluated in patients with COVID-19. These heterogeneous presentations might have different underlying mechanisms. Anosmia and hypogeusia seem to be distinct from two other clusters of neuropsychiatric symptoms, i.e., more general constitutional-like symptoms and more specific neuropsychiatric symptoms. Skeletal muscular manifestations might be a constitutional or a neuropsychiatric symptom.

## Methods

We evaluated 201 participants with a confirmed diagnosis of COVID-19 (52 outpatients and 149 inpatients) from March 2019 to April 2020 in a large referral Hospital in Tehran (Rasoul Akram Hospital). Demographic characteristics and comorbidities are shown in Table 2. The diagnosis was based on a positive real-time reverse transcription-polymerase chain reaction assay using a COVID-19 nucleic acid detection kit or a typical chest CT scan for COVID-19 reported by three specialists (an emergency medicine specialist, an infectious disease specialist, and a pulmonologist). The diagnosis was supported by related laboratory tests (biochemistry and inflammatory factors). Patients were diagnosed with COVID-19 according to WHO interim guideline^24^.

**Table 2:** Demographic characteristics and comorbidities in 201 patients with COVID-19.

Characteristics	Total	Male	Female
**Age, mean (SD), year**	51.84 (16.57)	51.36 (14.37)	52.54 (15.40)
**Weight, mean (SD), kg**	76.51 (18.27)	81.42 (20.10)	69.40 (12.2)
**Gender, number (percent %)**	201 (100%)	119 (59.2%)	82 (40.8%)
**Patient status, number (percent %)**			
Inpatients	142 (70.6%)	86	56
Outpatients	52 (25.9%)	29	23
ICU	7 (3.5%)	4	3
**Comorbidity, number (percent%), Yes**	107 (53.2%)	53	54
Diabetes mellitus	37 (18.4%)	21	16
Cerebrovascular disease	17 (8.4%)	5	12
Hypertension	28 (13.9%)	16	12
Pulmonary disease	10 (4.9%)	5	5
Renal disease	11 (5.4%)	6	5
Cancer	6 (2.9%)	2	4
Hyperlipidemia	7 (3.4%)	4	3
Endocrine disease	9 (4.4%)	3	6
Hematologic disease	6 (2.9%)	1	5
Rheumatologic disease	6 (2.9%)	1	5
EarNoseThroat disease	5 (2.4%)	3	2
Psychiatric disease	1 (0.49%)	1	0
Neurologic disease	5 (2.4%)	2	3

All of the patients or the closest available relative gave an informed consent for the study. This research has been approved by the ethics committee of the Iran University of Medical Science (code number: IR.IUMS.REC.1399.080).

### Data collection

Those with no or mild symptoms were quarantined at home and were followed to answer the online checklist. The follow-up was done by a trained medical student. Patients with moderate to severe symptoms were hospitalized and examined daily by a trained neurologic resident. We collected the data from conscious cognitively capable patients and their families as well as from the medical team where needed. Then, we documented the data including demographics, comorbidities, drug history, neuropsychiatric history, symptomatology of coronavirus infection. History of seizure or stroke during COVID-19 was confirmed by a related specialist, if reported. For hospitalized patients, chest CT Scan, laboratory tests including arterial blood gas analysis, count blood cell (CBC), liver function test, renal function test, inflammatory markers, creatine phosphokinase test (CPK) if requested for the patient were all documented. Criteria for additional laboratory tests were saturation sat O_2_ < 90%, impaired consciousness, systolic blood pressure < 90 MMHG, medical disease comorbidity, deterioration of the course the disease, dehydration and intolerance of oral intake or when necessary to diagnose or follow-up patients, therefore some inpatients did not have all paraclinical data.

### Statistical analysis

All statistical analyses were performed using R (version 3.6.1; R Core Team, 2019) in RStudio (RStudio-1.2.5001). For numerical variables, we used mean and standard deviation (SD) when they were normally distributed and we used range if they were not. Continuous variables were compared by using the Wilcoxon rank-sum test.

To perform a cluster analysis in R, we prepared data as rows were observations (individuals) and columns were variables, and we removed the missing values. We also used the “StatMatch” package to find the dissimilarity matrix, and we used the “cluster” package in clustering algorithms. We used the function “hclust”, and specified the agglomeration method as “ward.D2” method and we plotted the dendrogram (Fig. 2). Finally, we visualized the result in a scatter plot by utilizing the “factoextra “package (Fig. 3).

To find the predictor factors in our laboratory measures, we performed the stepwise regression by iteratively adding and removing variables in each step. To do this in R, we used “MASS” package, which choose the best model based on AIC criterion, and we used “stepAIC” function for stepwise regression (Table 1). The significance threshold at a 2-sided P value was set at less than 0.05.

### Ethics declarations

Written informed consent was obtained from all of the patients or the closest available relative for the study. This research has been approved by the Ethics Committee of the Iran University of Medical Science (Code Number: IR.IUMS.REC.1399.080).

## Data Availability

Corresponding author had full access to all of the data in this study and I take complete responsibility for the integrity of the data and the accuracy of the data analysis. The authors agree with sharing, coping, and modifying the data used in this article, even for commercial purposes, so long as appropriate credit is given, and possible changes are indicated.
